# Role of Rotated Head Postures on Volunteer Kinematics and Muscle Activity in Braking Scenarios Performed on a Driving Simulator

**DOI:** 10.1007/s10439-022-03087-9

**Published:** 2022-10-12

**Authors:** Fabian Kempter, Lorena Lantella, Norman Stutzig, Jörg Fehr, Tobias Siebert

**Affiliations:** 1 Institute of Engineering and Computational Mechanics, University of Stuttgart, Pfaffenwaldring 9, 70569 Stuttgart, Germany; 2 Institute of Sport and Movement Science, University of Stuttgart, Allmandring 28, 70569 Stuttgart, Germany

**Keywords:** Volunteer testing, Driving simulator, Neck musculature, Electromyography, Rotated head posture

## Abstract

Occupants exposed to low or moderate crash events can already suffer from whiplash-associated disorders leading to severe and long-lasting symptoms. However, the underlying injury mechanisms and the role of muscle activity are not fully clear. Potential increases in injury risk of non-nominal postures, i.e., rotated head, cannot be evaluated in detail due to the lack of experimental data. Examining changes in neck muscle activity to hold and stabilize the head in a rotated position during pre-crash scenarios might provide a deeper understanding of muscle reflex contributions and injury mechanisms. In this study, the influence of two different head postures (nominal vs. rotation of the head by about 63 ± 9° to the right) on neck muscle activity and head kinematics was investigated in simulated braking experiments inside a driving simulator. The braking scenario was implemented by visualization of the virtual scene using head-mounted displays and a combined translational-rotational platform motion. Kinematics of seventeen healthy subjects was tracked using 3D motion capturing. Surface electromyography were used to quantify muscle activity of left and right sternocleidomastoideus (SCM) and trapezius (TRP) muscles. The results show clear evidence that rotated head postures affect the static as well as the dynamic behavior of muscle activity during the virtual braking event. With head turned to the right, the contralateral left muscles yielded higher base activation and delayed muscle onset times. In contrast, right muscles had much lower activations and showed no relevant changes in muscle activation between nominal and rotated head position. The observed delayed muscle onset times and increased asymmetrical muscle activation patterns in the rotated head position are assumed to affect injury mechanisms. This could explain the prevalence of rotated head postures during a crash reported by patients suffering from WAD. The results can be used for validating the active behavior of human body models in braking simulations with nominal and rotated head postures, and to gain a deeper understanding of neck injury mechanisms.

## Introduction

Automated driving enables non-driving activities and thus lead to changes in occupant postures and positions compared to conventional driving.^5^ Even in current traditional driving conditions, the occupants show rotated or tilted head postures in considerable parts of the time.^9,34^ This affects the stress state of the musculoskeletal system and influences the behavior of the occupant in driving and crash scenarios. The associated influences on the injury risk have only been merely investigated.

Non-nominal (i.e. rotated or tilted) head postures are assumed to show a higher risk of suffering soft-tissue injuries, such as whiplash-associated disorders (WAD) in low-velocity impacts. Based on self-reporting data, turning the head in such a scenario increases the risk by 50% compared to looking straight ahead.^16^ Also, MRI-verified studies observed increased ligament damages in WAD patients who reported non-nominal head postures during crashes.^18^ In addition, patients with chronic whiplash reported more head rotation and tilting at the time of impact than asymptomatic patients (46–57% vs. 24–28%).^33,39^

Muscle activity is assumed to be important in such accident scenarios, as the muscles are sufficiently activated during the relevant phases of the accident to affect whiplash injury mechanisms.^26,36^ However, it is not clear if muscle activity mitigates or aggravates whiplash injuries in typical car crashes.^37^ Differences in initial muscle activity in contrast may affect the entire duration of the scenario, while muscle reflexes suffer from intrinsic delays. Virtual Human Body Models (HBMs) can be used in such investigations and can help to create a better understanding of the injury mechanism in WAD.^7,19,30,32^ Because the HBMs allows the measurements of intrinsic values like ligament strain, muscle state, or pressure in the intervertebral discs, which are almost impossible to obtain in human experiments due to ethical aspects. Volunteer tests play an essential role in the calibration and validation process of HBMs, as they allow matching the controlled HBM kinematics with observed human behavior.^8,15,17,32^ Additionally, the active neuromuscular behavior of subjects using surface electromyography can help to define appropriate muscle controller with improved physiological accordance.

The role of muscle activity in WAD injury mechanisms is not fully understood. During a crash, various stimuli could trigger the cervical spine musculature, such as subject kinematics, vehicle motion, acoustic signals, or vibrations. Various reflexes might contribute to the muscular behavior of the subject, e.g., postural responses mediated by more distal mechanoreceptors, the vestibular reflex, the startle reflex, or stretch reflexes of neck muscles.^26^ Especially the latter one might be relevant for avoiding injuries (e.g., whiplash) because stretch reflexes might increase muscle activity during the impact situation. For example, during impacts the trapezius muscles will be stretched, resulting in excitation of their muscle spindles. Due to the monosynaptic connection between afferent Ia fibers and alpha motoneurons of the homonymous muscle, the muscle will be activated. The muscle onset times during the stretch reflex depend on the length of the reflex circuit.^31^ In experiments with low or moderate braking excitations,^22,26^ averaged muscle onset times in the range of 150 to 170 ms for trapezius (TRP) and 150 to 370 ms for sternocleidomastoideus (SCM) were reported. In Ref. 22, the authors reported an increase in the muscle onset times when the excitation level was reduced.

Sled tests and experiments in real cars were frequently used to examine muscle activation and passenger movement in impact or braking situations. In different studies, acceleration pulses were applied in sled tests with variations of pulse intensity, direction and whether the subjects are informed at the time of pulse application, e.g. Refs. 2,4,6,21,28. Investigations performed inside real vehicles studied effects of belt-pretensioning and combined maneuvers.^11,13,29^ While the influence of increased muscle pre-activation due to anticipation has often been studied, asymmetrical initial muscle states due to rotated head postures are only rarely addressed in this type of experiments.^23,24^

The influence of visual feedback of a crash scenario combined with mechanical perturbation is not, or only rarely, investigated. In standard sled tests, subjects do not perceive any visual pre-crash event but only see the laboratory environment. This might influence the muscle preactivation, body posture and subjects’ behaviour. Consequently, different conditions of the subjects (aware vs. unaware or relaxed vs. braced) can only be induced by giving information about the start of the sled movement (e.g. by a countdown), or by actively adopting a pretensed posture. In contrast, in driving simulators the subjects can experience a virtual reality scenario^14^ while exposed to reproducible external loads in a safe environment.

The objectives of this study are to investigate the influence of rotated head postures on the kinematics and muscle activity of relevant neck muscles compared to nominal head postures during a virtual braking event.

## Materials and Methods

### Subjects

Seventeen healthy subjects participated in the experiment. Three female and fourteen male subjects (body mass: 72 ± 10 kg, age: 24 ± 2 years, body height 180 ± 12 cm, and body mass index of 22 ± 2). All subjects were informed about the aims and risks of the study and gave their written consent to participate. The study was approved by the ethical committee of the University of Stuttgart (AZ19-001) and was conducted in accordance with the latest declaration of Helsinki.

### Experimental Setup and Procedure

#### Setup of the Driving Simulator

**Figure 1:**
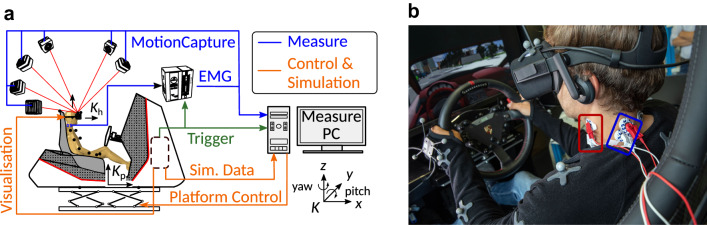
Driving Simulator Setup. Left: Signal flows of the driving simulator and measuring hardware with coordinate systems of platform *K*_p_ and head *K*_h_. Right: Subject inside the driving simulator with electrode placement on the muscles TRP (blue) and SCM (red), (c) University of Stuttgart/Uli Regenscheid. The gray reflective markers for motion capturing (right) are displayed as black circles in the left picture. Note: In the experiments presented here, the subject’s hands are not on the steering wheel, but on the legs.

The driving simulator consists of a 6-degree of freedom Stewart platform CKAS W3s 6DOF motion system with maximal motion capacities (translational: **s**_lim_ =  ± 5 cm, $\dot{{\varvec{s}}}$_lim_ =  ± 10 cm*/*s, $\ddot{{\varvec{s}}}$_lim_ = 0*.*3 g; rotational: $\varphi$ =  ± 10°, $\dot{\varphi }$=  ± 15°*/*s, $\ddot{\varphi }$ =  ± 150°*/*s^2^) and a Porsche GT3 mock-up, see Fig. 1. The visualization is ensured by Oculus Rift head-mounted displays (HMD, weight: 470 g) and virtual desktop app that provides interactive visualization based on the position and orientation of the subject’s head. The virtual scenario was modelled using Simcenter PreScan version 8.2.0. A Simulink model was generated to control the virtual crash event, to activate the platform and to synchronize the motion capture system and the EMG system. A more detailed description is given in Ref. 20.

To analyze the drivers’ behavior in the driving simulator, the muscle activation was measured using surface electromyography and kinematics was measured using a motion capture system.

#### Electromyography

Bipolar surface electromyografic (SEMG) data were recorded from the left and right sternocleidomastoideus muscles (SCM_l and SCM_r) and the upper part of the trapezius muscles (TRP_l and TRP_r) using the Biopac MP160 system (Biopac Systems, Goleta, California, USA). SEMG electrodes were located based on the recommendations of Ref. 1. To reduce skin resistance and to improve skin conductivity, the corresponding skin sites were prepared according to the guidelines of SENIAM^12^ before attaching the electrodes. The first step was to thoroughly remove the hair using a disposable razor. Subsequently, the skin was roughened and cleaned with disinfectant sprays. Then two bipolar electrodes were attached on each muscle with an inter-electrode distance of 20 mm. The reference electrode was positioned on the acromion.

#### Kinematics

Subject kinematics were recorded using a Motion capture system (OptiTrack Prime, NaturalPoint Inc., Corvallis, OR, USA) consisting of six infrared cameras (4 × Prime 13 W and 2 × Prime^x^ 13) mounted stationary in the laboratory. A frame rate of 200 fps was used. Spatial position reconstruction and triggered recording were performed with the corresponding MOTIVE Tracker software version 2.2.0. The calibration procedure of the OptiTrack system was performed before each experiment group (minimum once per day) and resulted a mean error of 0.3 to 0.4 mm. In relation to the mean head displacement of 20 mm, the mean error is about 2%. Before the SEMG electrodes were plugged in, the subjects put on a motion capture suit to analyze upper body motion. Reflexive markers were placed at the upper extremities and the distal and proximal end of the subject’s sternum. The subjects were asked to put on the HMD equipped with three additional markers (Fig. 1, right). This ensures the tracking of head translation and orientation during the experiments without prior skeleton reconstruction or manual marker assignment. The center location of the virtual object defined by the HMD markers was translated to the head center of gravity inside the MOTIVE software. This vector used to offset the point of interest was assumed to be time-invariant (no relative motion between HMD and head) and constant for all subjects without accounting for anthropometric variability between subjects.

#### Synchronisation

The OptiTrack- and Biopacsystem were started by an external trigger, provided by the environment simulation of the driving simulator. This software trigger was translated into an electrical signal using an Arduino Uno Rev3 for SEMG triggering, and a udp trigger signal for the pre-initialized connection to the OptiTrack Natnet SDK.

### Procedure and Protocol

#### Test Procedure

**Figure 2:**
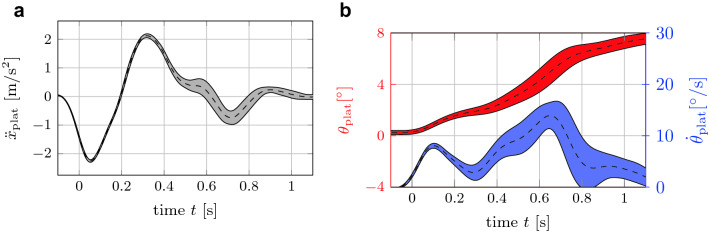
Platform motion. Platform motion during braking event with longitudinal acceleration  ${\ddot{x}}_{plat}$ (left, gray), pitch rotational displacement ${\theta }_{plat}$ (right, red) and pitch rotational velocity  ${\dot{\theta }}_{plat}$ (right, blue). Mean values (dashed) ± 1 standard deviation (corridors). Note that deviations in platform motion despite similar driving scenarios appeared due to platform loading resulting from interindividual differences in subjects’ weight and height.

Following skin preparation and marker positioning, subjects performed two maximal isometric voluntary contractions (MVC) against the hand of the experimental leader each for neck flexion and neck extension with 30 s rest between. Subsequently, the subjects were exposed to an automated driving scenario with fully automated braking events while sitting as an inactive driver with their hands on their legs. The virtual car controlled its speed and direction fully autonomously resulting in high reproducibility and allowing the driver to act like a passenger without any driver inputs. The virtual braking event was represented by the platform motion, where the x-translation and the rotation around the y-axis (pitch motion) were calculated with a motion queuing algorithm, see Fig. 2. The platform was initially located to the front of its maximum motion range and accelerated backwards to represent the braking pulse. The initiated backward motion must be stopped by positive accelerations in order not to violate the maximum motion range. In addition, a pitch motion was applied to emulate low-frequency components of the acceleration signal, see Fig. 2 on the right.

Each subject performed three runs of each of the variants in a random order according to a pre-generated protocol. In the variants presented in this study, only the head orientation (nom vs. rot) was varied, see Table 1. The platform movement remained unchanged for all variants. In the scenarios with rotated head, the subjects were asked to perform and hold a shoulder view to the right without moving their upper body. The desired head posture was additionally presented by the experimental leader.

**Table 1:** Overview of the variants performed in the study varying the head orientation.

Variant	Head orientation	Visual feedback	Takes per subject
Nom	Nominal	HMD	3
Rot	Right shoulder check	HMD	3

#### SEMG Processing

**Figure 3:**
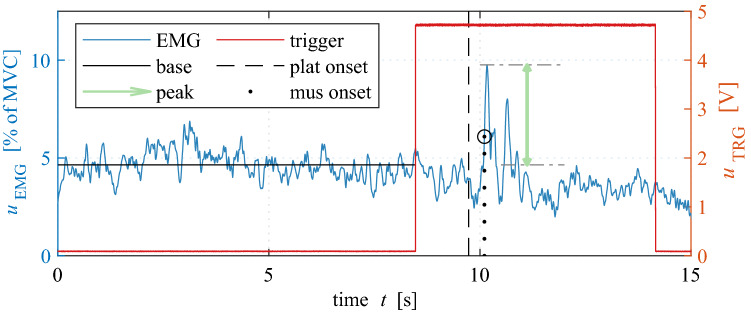
SEMG processing. Processing SEMG signals *u*_EMG_ (blue) to obtain base activity (base, black) and peak activation value (peak, green) exemplarily shown on a SCM_l signal displayed as percentage of MVC in a right shoulder check experiment (rot) with trigger signal *u*_TRG_ (red) used for synchronizing EMG with kinematic data. Visualization of the onset times of platform motion (plat onset (platform onset), black dashed) and muscle activation (mus onset (muscle onset), black dotted).

All SEMG signals were recorded with a sampling frequency of 2 kHz, preamplified by a factor of 2000, filtered from 10 to 500 Hz using a bandpass filter and stored to a computer.^40^ Subsequently, the signals were processed by rectifying, and smoothing the signals using a moving average of ± 100 samples. The SEMG signals were normalized to the maximum amplitude of the MVC measurements,^41^ which can be interpreted as the degree of muscle activation. To determine the muscle onset time, the method of Hodges and Bui was used.^38^ For this purpose, the onset was defined as the first sample where the signal was three standard deviations above the baseline activity. The baseline activity was calculated as the mean value from the beginning of the measurement to the time when the synchronisation signal surpassed the 1 V threshold. The onset time was calculated from the start of the braking maneuver, which equals the motion onset of the platform, to the muscle onset. If no onset could be determined in the phase until 500 ms after platform onset, the test was not included in the evaluation. The reflex amplitude was determined according to Ref. 26 by first determining all local maxima and minima within 20 ms to 300 ms after the muscle onset. The first local maximum was then searched, which was not followed by a local minimum for more than 10 ms. If there were two separate reflex components, only the first was considered. This local maximum was divided into a baseline activity (base) before pulse application and a peak value (peak) to differ between activity induced by holding the desired posture and additionally induced activity by external excitation, see Fig. 3.

#### Kinematics Processing

The kinematics of the subject in the inertial system were transformed into the moving, body-fixed coordinate system of the simulator. The derivates of the kinematic data were obtained by filtering the position data using a 4th order low-pass Butterworth filter with a cutoff frequency of 2.5 Hz, subsequent cubic spline interpolation and derivation of the resulting function in Matlab R2019b. Motion onsets of the platform and the body segments were determined using velocity thresholds. The kinematic thresholds were set to 10% of their maximal velocity in the initial phase. The time axis was shifted so that the beginning of the platform onset is defined as *t* = 0. All other onsets indicate the time elapsed after the platform’s onset criterion was met.

#### Statistical Analysis

All data are presented as means with standard deviations (SD). Wilcoxon signed rank tests were performed to investigate the effect of altered head postures, i.e., nominal vs. rotated, on the SEMG signals, i.e., base activation, peak activation, and onset times. The resulting significance level *p* is categorized into moderate (*, *p* < 0*.*05), strong (**, *p* < 0*.*01), very strong (***, *p* < 0*.*001). The statistical analyses were performed using IBM SPSS Statistics for Windows (Version 27.0., IBM Corp, Armonk, NY, USA).

#### Excluded Data

Due to technical problems with the platform motion, the results of one subject were excluded from this study, resulting in a group of two female and fourteen male subjects (body mass: 73 ± 10 kg, age: 24 ± 2 years, body height 181 ± 11 cm, and body mass index of 22 ± 2). In addition, muscle data from TRP_r of subject YU948 were discarded due to incorrect MVC values, resulting in activity values of above 100% and highly increased compared to the TRP_l in the nominal posture.

## Results

### Kinematics

**Figure 4:**
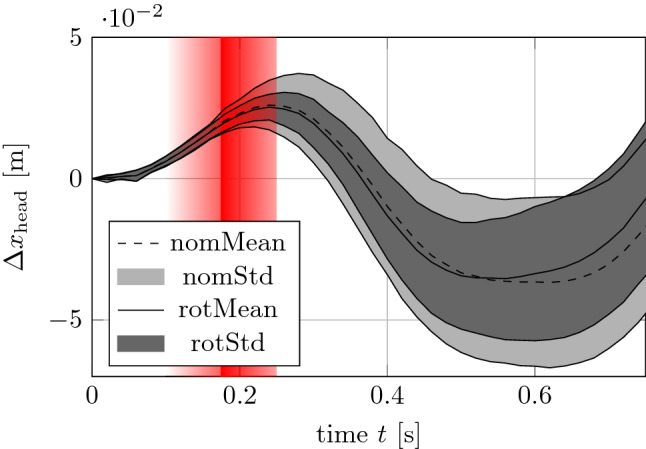
Kinematic Results. Head relative x-displacement ∆*x*_head_ displayed in the platform coordinate system *K*_p_. Mean values (lines) and standard deviation for nominal (light gray area) and rotated head posture (dark gray area). The red shaded area marks the time range, where physiological reflexes are expected. *t*_0_ = 0: platform motion onset.

In the scenarios with rotated head, the participants adopted postures with a mean yaw angle of − 63° (± 9°). The mean head x-displacement showed similar characteristics for both head orientations (nom vs. rot). In the first phase after the platform motion onset (*t*_0_ = 0), the head shows forward displacement with a moderate variance. After the maximum forward displacement (27*.*5 ± 10*.*1 mm) was reached at *t* = 248 ± 30 ms, the subjects moved their head back to the starting position at *t* = 376 ± 41 ms (Fig. 4). In the following phase, the standard deviation of the head displacement increased. However, the standard deviation in rotated head postures was lower compared to the nominal head posture (Fig. 4).

**Figure 5:**
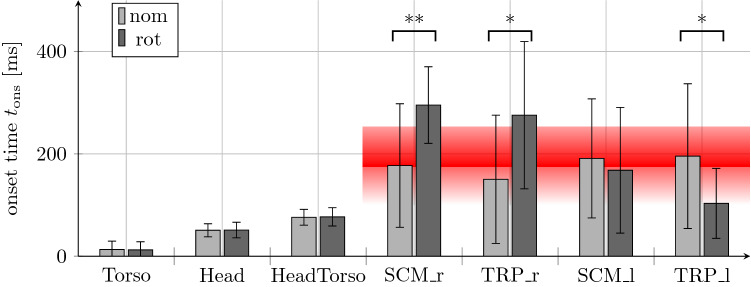
Onset times. Motion and muscle onset times as mean values (bar plot) and standard deviation (lines). Red shaded area marks time corridor of potential reflexes triggered by platform motion or body segment displacements. *t*_0_ = 0: platform motion onset.

The **kinematic onsets** of body segments after *t*_0_ show no significant difference between the head postures. The mean torso movement started at 15 ms shortly after the platform motion onset. The onset of head movement and the head movement relative to the torso followed on average at 50 ms and 75 ms, respectively (Fig. 5).

### Muscle Activation

**Figure 6:**
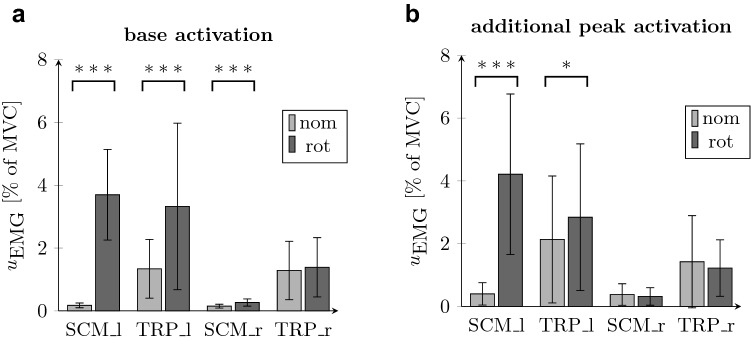
SEMG amplitudes. EMG levels in % of MVC displayed as mean values (bars) and standard deviation (lines) for nominal (light gray) and rotated (dark gray) view. (a): base activation (left) (b): additional peak activation *u*_EMG*,*rflx_ − *u*_base_ (right). *t*_0_ = 0: platform motion onset.

As expected, holding the head in a nominal position required similar activations of both contralateral SCM muscles and of both contralateral TRP muscles (Fig. 6 left). However, required muscle activity was on average eight times higher in TRP compared to SCM in the nominal position. The SEMG data of SCM and TRP demonstrate significant changes in base activation as well as in peak activation induced by head rotation, see Fig. 6. Base activation showed significantly increased values during rotated head postures for the muscles on the left side SCM_l (*z* = *− *6*.*03*, p* < 0*.*001) and TRP_l (*z* = *− *5*.*72*, p* < 0*.*001). The muscle SCM_r (*z* = *− *5*.*88*, p* < 0*.*001) showed a significant increase, while keeping at lower level of activation. TRP_r showed no significant difference (*z* = − 1*.*01*, p* = 0*.*312). Peak activation values showed significant changes only in the muscles SCM_l (*z* = *− *5*.*07*, p* < 0*.*001) and TRP_l (*z* = − 2*.*03*, p* = 0*.*042) between both investigated head postures.

The **muscle onsets** in the nominal head posture showed similar values for all muscles in the range of 125 ms to 200 ms. In the rotated head posture, the values of the contralateral left muscles SCM_l (*z* = *− *3*.*17*, p* = 0*.*002) and TRP_l (*z* = *− *2*.*50*, p* = 0*.*012) are increased, while the muscle onset time of TRP_r (*z* = *− *1*.*99*, p* = 0*.*046) was decreased. The muscle SCM_r (*z* = *− *0*.*42*, p* = 0*.*672) showed no significant difference between the investigated groups (Fig. 5). In approximately 10% of SCM signals, and 35% of the TRP signals, respectively, the algorithm used for muscle onset detection did not detect any muscle onset.

**Figure 7:**
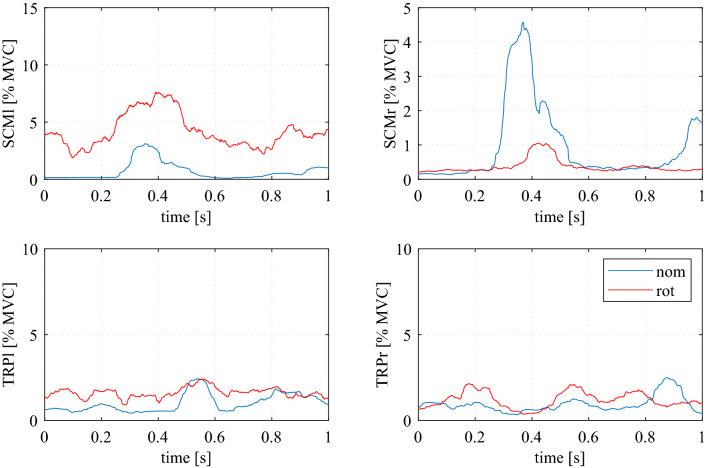
EMG signals of subject HU456 for nominal (blue) and rotated head posture (red), one trial each.

**Figure 8:**
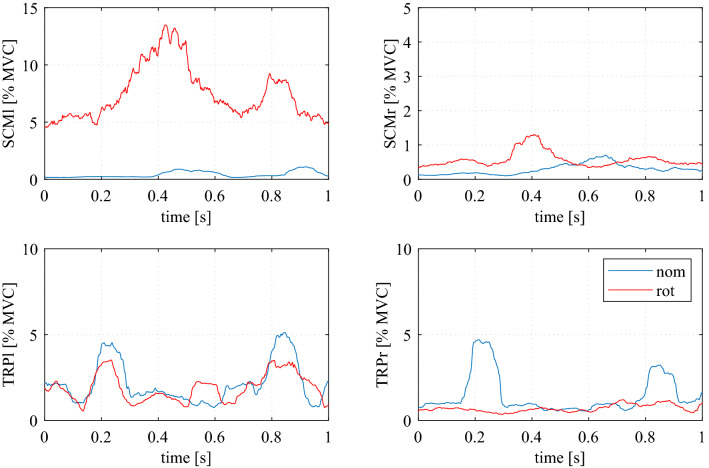
EMG signals of subject MT825 for nominal (blue) and rotated head posture (red), one trial each.

**Figure 9:**
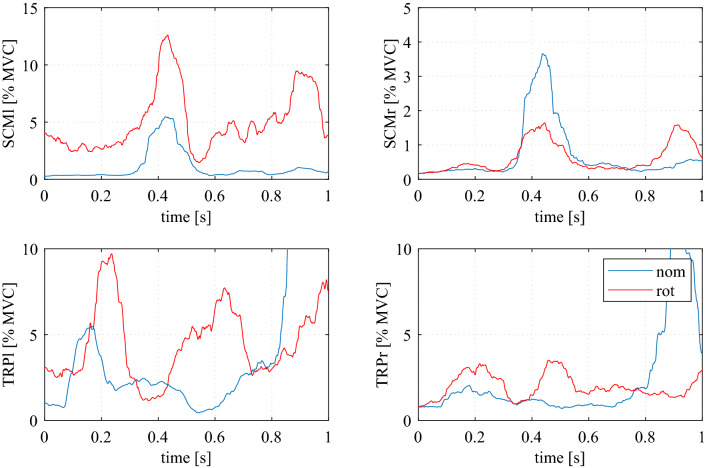
EMG signals of subject MZ731 for nominal (blue) and rotated head posture (red), one trial each.

In Figs. 7, 8 and 9, EMG signals of three volunteers are exemplarily shown for one trial with nominal and rotated head posture.

## Discussion

We examined the impact of head position on muscle activity and head kinematics during simulated braking events in a driving simulator with visual feedback. Compared to Ref. 24, our study examined rotated head postures with higher yaw angles (63° vs. 45°). In our study, the subjects showed similar kinematic behavior in stabilizing the head under the external excitation applied by the motion platform independently from their head posture. The motion of the head in backward x-direction after initial head forward motion is assumed to be triggered by reflexive muscle activity and positive acceleration values of the platform which were needed to stop the platform from violating the maximum motion space. The general head displacement of about 30 mm (Fig. 4) is lower than in other experiments replicating braking or frontal crash events. This can be explained by the lower level of the platform excitation as well as in the shorter duration of the negative braking pulse which is followed by a phase of acceleration in positive x-direction. To mitigate the effect of this change of direction in the translational platform excitation, pitch motions commonly used in the field of Motion Cueing^27^ were superposed. The usage of platform pitch motion to imitate translational accelerations in biomechanical investigations is motivated by a study presented in Ref. 10, where seated volunteers showed similar muscle activation patterns when exposed to forward translational acceleration or legged-up tilting of the motion platform. With this additional applied kinematic excitation, the subjects perceived increased acceleration values over a longer period of time and therefore their effort in stabilizing their bodies is supposed to be increased compared to purely translational movements of the platform.

We examined changes in muscle onset times, base and peak activation. The execution of simulated braking events in a driving simulator with visual feedback resulted in neck muscle activities depending on the muscle (SCM vs. TRP) and head posture (nominal vs. rotated).

Different head positions (nom vs. rot) clearly led to different muscle activation values. The increase of muscle activity of the contralateral left SCM_l in the base activation (Fig. 6 left) was expected as increased SCM_l activity is required to keep the head in the right rotated position. Together with the increased peak values of SCM_l during rotated head posture (Fig. 6, right), our observations of asymmetrical muscle acitivity are in accordance with results of frontal sled tests with variations of rotated heads shown in Refs. 22,23. However, in Refs. 22,23 the EMG signals showed clearly higher values of up to 75% of MVC and are presented as total muscle activity without a separation of static (posture-dependent) and dynamic (reflex-triggered) effects. Higher levels of muscle activation result from higher acceleration scenarios in their study (*a* = 4*.*2 to 13*.*0 m*/*s^2^) using a sled with a pneumatic cylinder instead of a hexapod platform. In our study with relatively low magnitudes and shorter application durations of the platform excitation, the base and peak activation values were found to be in a similar range with approximately half of the total muscle activity. By separating static and dynamic components of the EMG signals further insights were gained that can support the process of simulating human body models in rotated head postures by providing information about the physiological baseline condition of the subjects on the one hand and their reflex behavior during the braking scenario on the other hand. The contralateral left muscles (SCM_l, TRP_l) showed increased activity in base activation (Fig. 6, left) and peak activation (Fig. 6, right). Consequently, these muscles were required for holding the head in rotated position and for head stabilization during the braking event. The platform motion is supposed to induce additional yaw torques on the rotated head which need to be counteracted by the neck musculature leading to increased peak activation in the muscles responsible for rotating the head rotation before. In contrast, right muscles (SCM_r, TRP_r) had much lower activations and showed no changes in muscle activation between nominal and rotated head position except for SCM_r which yielded slightly increased base muscle activation from 0.15 to 0.27% of MVC. This increase in very small absolute values can be considered irrelevant for injury risk assessment. Thus, the right muscles play a minor role in head positioning and stabilization in the scenario with the head rotated to the right (rot).

The muscle onsets were in an expected range where physiological reflexes are reasonable sources of activation. Only the preactivated muscles SCM_l and TRP_l (marked by increased base activation; Fig. 6 left) in the rotated posture showed increased onset times above the expected time corridor of potential reflexes (Fig. 5, red shaded area). The onsets could be triggered by multiple stimuli, such as kinematic excitation by head rotation, torso acceleration or muscle-spindle reflex due to relative head to torso motion. The tendency for muscle onset times to be at the upper end of the expected reflex range might be associated with the relatively low kinematic excitation during the scenario. Compared to other experiments with low or moderate braking excitations^22,26^ averaged muscle onset times in the range of 150 to 170 ms for TRP and 150 to 370 ms for SCM were reported. In accordance to Ref. 22, our study shows increased values of muscle onset times while using a lower level of mechanical excitation by the experimental setup compared to Refs. 22,26. In addition, the values are displayed in relation to the motion onset of the platform. However, some physiological reflexes are triggered not by the platform motion itself, but by deformations or relative displacements, such as the elongation of the neck. The start of this elongation can be characterized by the motion onset of the head motion relative to the torso, which started about 75 ms after the motion onset of the platform (Fig. 5). Signals where no muscle onset could be detected indicate very low peak muscle activity or suboptimal signal quality covering low muscle peak values.

In contrast to Ref. 23, the EMG data presented here suggest a significant delay in the muscleonset times of the contralateral left muscles in the rotated head posture. These differences between both studies could be explained by differences in the scenario by means of a more rotated head posture, the usage of a VR headset and a different type of excitation with pitch motion and lower level of acceleration. In addition, using a driving simulator instead of a sled could affect the anticipation of the volunteers, which might result in changes in neuromuscular reaction, e.g., onset times. The increase in the muscle onset time could be explained by several effects. One possible source of increased onset times is the higher initial activation level of SCM_l and TRP_l. In accordance to Ref. 26, the preactivated muscles in an antagonistic-agonistic setup showed delayed onset times than in the nominal posture or relaxed state. The higher base activation of TRP_l in the rotated head posture could lead to a reciprocal inhibition of the *α*-motor neurons in the antagonistic SCM_l, which could explain the higher onset times of SCM_l.^26^ This applies in the same way to the onset times of the TRP_l by the increased base activation of SCM_l. Another possible effect for longer muscle onsets might be due to the different point of view during driving scenarios with rotated head postures. This changes the attention of the subject. Various important situational features that trigger muscle activation might be perceived. This might lead to longer muscle onsets during braking or crash events. Besides the physiological explanations, also methodological effects in the process of muscle onset detection in signals with higher base activation could lead to increased muscle onset times as well, e.g., by increased SD levels used in the motion onset detection.

Considering that decreased muscle activity is associated with lower stiffness, delayed muscle onset times and the thereby reduced muscle activity could lead to less neck stability in case of a crash. However, the rotated head with increased muscle base activity might already result in a higher stiffness and prestress in some muscles, compared to the nominal posture. The asymmetrical muscle state, i.e., strain^35^ and activity, prior and during the braking scenario could affect the risk of injury, e.g., by increased force capacity of pre-activated muscles^25^ known as active lengthening,^43^ or by changing force capacity depending on muscle length, stretch amplitude and stretch velocity.^42,44^ Simulations based on the here reported results could help to investigate effects of asymmetrical postures and the corresponding muscle states on muscle and ligament loading. However, additional studies are needed to investigate these effects in a realistic scenario with higher level of kinematic excitation.

### Limitations

The chosen experimental setup provides only limited kinematic excitation which is not directly transferable to motions experienced in realistic road vehicle driving situations. The additional applied pitch motion probably triggers human reaction which do not necessarily match with the reactions seen in real-world traffic and cannot adequately replicate the lack of longitudinal acceleration capabilities of the platform. Due to the limited motion range of the platform, the platform cannot be slowed down smoothly from the accelerated motion replicating the braking pulse. The abrupt stop of the backward motion by the platform lead to positive acceleration values which are characteristic for rear-end impacts. The usage of head-mounted displays results in increased inertia in the head and a forward shift of its center of gravity. The additional mass of 470 g (10% of head mass) affects the task of the neck muscles to keep the head stable under external excitation. The increased immersion by omitting the perception of the laboratory surrounding was rated higher than the waiver of additional inertia in this study. In addition, HMDs are supposed to be used more often in future vehicle concepts, e.g., for entertainment or to prevent motion sickness. The tests were nonetheless carried out in a laboratory environment and thus still have limitations in terms of immersion. A limitation of the study is that female participants are underrepresented in the sample. It was reported in the literature that female participants have a somewhat higher relative risk of whiplash injury than males.^3^ This might be associated with gender specific differences in muscle onset times during car accidents. The muscle MVC levels were obtained in the nominal posture only, which limits the level of detail for the absolute values of muscle activity in the rotated head posture scenarios.

### Conclusion

In this study, the influence of rotated head postures was investigated in virtual braking experiments inside a driving simulator. The subjects demonstrated similar kinematic behavior in stabilizing the head under the external excitation applied by the motion platform independently from their head posture. In contrast, the muscle onset time, peak and base muscle activation showed significant differences in rotated head posture compared to nominal head posture. The separation of base and peak activation revealed more insight especially when investigating different postures requiring a certain level of effort. In scenarios with low or moderate excitation, the base activation could be in a similar range of the actual dynamic muscle response (peak) and therefore could cover potential phenomena. Therefore, this study emphasizes a separation of static and dynamic effects in EMG data when investigating non-nominal postures with low external excitation. The results could be relevant for the validation of human occupant simulation models investigating the role of asymmetric physiological states in the biomechanical processes of injury leading, for example, to whiplash-associated disorders.

## Appendix

In Figs. 7, 8 and 9, EMG signals of three volunteers are exemplarily shown for two takes nom (blue) and rot (red) to provide insight in progression of muscle activity over time.

## References

[1] Barbero, M., R. Merletti, and A. Rainoldi. Atlas of Muscle Innervation Zones. Understanding Surface Electromyography and Its Applications. 2012.

[2] Beeman S, Kemper A, Madigan M, Franck C, Loftus S (2012). Occupant kinematics in low-speed frontal sled tests: human volunteers, hybrid III ATD, and PMHS. Accid. Anal. Prev..

[3] Berglund, A., L. Alfredsson, I. Jensen, L. Bodin, and A^˜^. Nygren. Occupant- and crash-related factors associated with the risk of whiplash injury. *Ann. Epidemiol.* 13:66–72, 2003. 10.1016/s1047-2797(02)00252-1 12547487

[4] Blouin J-S, Siegmund GP, Inglis JT (2007). Interaction between acoustic startle and habituated neck postural responses in seated subjects. J. Appl. Physiol..

[5] Bohman K, Örtlund R, Groth GK, Nurbo P, Jakobsson L (2020). Evaluation of users’ experience and posture in a rotated swivel seating configuration. Traffic Injury Prev..

[6] Choi, H. Y., S. Sah, B. Lee, S. J. Kang, M. S. Mun, I. Lee, and J. Lee. Experimental and numerical studies of muscular activations of bracing occupant. In: 19th Enhanced Safety of Vehicles, Washington, DC2005.

[7] Correia MA, McLachlin SD, Cronin DS (2021). Vestibulocollic and cervicocollic muscle reflexes in a finite element neck model during multidirectional impacts. Ann. Biomed. Eng..

[8] Devane K, Johnson D, Gayzik FS (2019). Validation of a simplified human body model in relaxed and braced conditions in low-speed frontal sled tests. Traffic Injury Prev..

[9] Fice JB, Blouin J-S, Siegmund GP (2018). Head postures during naturalistic driving. Traffic Injury Prev..

[10] Forssberg H, Hirschfeld H (1994). Postural adjustments in sitting humans following ex- ternal perturbations: muscle activity and kinematics. Exp. Brain Res..

[11] Ghaffari, G., K. Brolin, D. Brase, B. Pipkorn, B. Svanberg, L. Jakobsson, and J. Davidsson. Passenger kinematics in lane change and lane change with braking manoeuvres using two belt configurations: Standard and reversible pre-pretensioner. In: Proceedings of the IRCOBI Conference, IRC-18-80, pp. 493–511, Athens, Greece, 2018.

[12] Hermens HJ, Freriks B, Disselhorst-Klug C, Rau G (2000). Development of recommen- dations for semg sensors and sensor placement procedures. J. Electromyogr. Kinesiol..

[13] Huber, P., S. Kirschbichler, A. Prüggler, and T. Steidl. Passenger kinematics in braking, lane change and oblique driving maneuvers. In: Proceedings of the IRCOBI Conference, pp. 783–802, Lyon, France, 2015.

[14] Ihemedu-Steinke, Q. C., R. Erbach, P. Halady, G. Meixner, and M. Weber. Virtual Re- ality Driving Simulator Based on Head-Mounted Displays, pp. 401–428, Cham: Springer, 2017.

[15] Iwamoto M, Nakahira Y, Kimpara H (2015). Development and validation of the total human model for safety (THUMS) toward further understanding of occupant injury mechanisms in precrash and during crash. Traffic Injury Prev..

[16] Jakobsson L, Isaksson-hellman I, Lindman M (2008). Whips (volvo cars’ whiplash protection system)—the development and real-world performance. Traffic Injury Prev..

[17] John J, Klug C, Kranjec M, Svenning E, Iraeus J (2022). Hello, world! VIVA+: A human body model lineup to evaluate sex-differences in crash protection. Front. Bioeng. Biotechnol..

[18] Kaale BR, Krakenes J, Albrektsen G, Wester K (2005). Head position and impact direction in whiplash injuries: associations with mri-verified lesions of ligaments and membranes in the upper cervical spine. J. Neurotrauma.

[19] Kato, D., Y. Nakahira, N. Atsumi, and M. Iwamoto. Development of human-body model thums version 6 containing muscle controllers and application to injury analysis in frontal collision after brake deceleration. In: Proceedings of the IRCOBI Conference, pp. 207–223, Athens, Greece, 2018.

[20] Kempter, F., J. Fehr, N. Stutzig, and T. Siebert. On the validation of human body models with a driver-in-the-loop simulator. In: The 5th Joint Int. Conference on Multibody System Dynamics (IMSD), Lisbon, Portugal, 2018.

[21] Kirschbichler, S., W. Sinz, A. Pru¨ggler, P. Huber, and K. Steiner. Detailed analysis of 3d occupant kinematics and muscle activity during the pre-crash phase as basis for human modeling based on sled tests. In: Proceedings of the 22nd International Technical Conference on the Enhanced Safety of Vehicles, pp. 1–8, Washington, D.C., 2011.

[22] Kumar S, Ferrari R, Narayan Y (2005). Kinematic and electromyographic response to whiplash loading in low-velocity whiplash impacts—a review. Clin. Biomech..

[23] Kumar S, Ferrari R, Narayan Y (2005). Kinematic and electromyographic response to whiplash-type impacts. Effects of head rotation and trunk flexion: Summary of research. Clin. Biomech..

[24] Kumar S, Ferrari R, Narayan Y (2005). Turning away from whiplash. An emg study of head rotation in whiplash impact. J. Orthop. Res..

[25] Minozzo, F. C. and D. E. Rassier. Effects of blebbistatin and ca2+ concentration on force produced during stretch of skeletal muscle fibers. Am. J. Physiol. Cell Physiol., 2010. 10.1152/ajpcell.00073.2010 20720178

[26] Mühlbeier A, Boström KJ, Kalthoff W, de Lussanet MHE, Kraaijenbrink C, Hagenfeld L, Castro WHM, Wagner H (2018). Neck muscle responses of driver and front seat passenger during frontal-oblique collisions. PLOS ONE.

[27] Nahon MA, Reid LD (1990). Simulator motion-drive algorithms—a designer’s perspec- tive. J. Guid. Control Dyn..

[28] Ólafsdóttir JM, Brolin K, Blouin J-S, Siegmund GP (2015). Dynamic spatial tuning of cervical muscle reflexes to multidirectional seated perturbations. SPINE.

[29] Ólafsdóttir, J. M., J. Östh, J. Davidsson, and K. Brolin. Passenger kinematics and mus- cle responses in autonomous braking events with standard and reversible pre-tensioned restraints. In: Proceedings of the IRCOBI Conference, IRC-13-70, pp. 602–617, Gothenburg, Sweden, 2013.

[30] Östh J, Mendoza-Vazquez M, Sato F, Svensson MY, Linder A, Brolin K (2017). A female head-neck model for rear impact simulations. J. Biomech..

[31] Pierrot-Deseilligny E, Burke D (2012). The Circuitry of the Human Spinal Cord: Neuro-plasticity and Corticospinal Mechanisms.

[32] Putra, I. P. A., J. Iraeus, F. Sato, M. Y. Svensson, A. Linder, and R. Thomson. Optimization of female head-neck model with active reflexive cervical muscles in low severity rear impact collisions. Annals of Biomedical Engineering, 2020. 10.1007/s10439-020-02512-1 PMC7773618 32333133

[33] Radanov, B. P., M. Sturzenegger, and G. Di Stefano. Long-term outcome after whiplash injury: A 2-year follow-up considering features of injury mechanism and somatic, radi- ologic, and psychosocial findings. Medicine 74, 1995. 10.1097/00005792-199509000-00005 7565068

[34] Reed MP, Ebert SM, Jones MLH, Hallman JJ (2020). Prevalence of non-nominal seat positions and postures among front-seat passengers. Traffic Injury Prev..

[35] Shateri H, Cronin DS (2015). Out-of-position rear impact tissue-level investigation using detailed finite element neck model. Traffic Injury Prev..

[36] Siegmund G, Brault J, Chimich D (2002). Do cervical muscles play a role in whiplash injury?. J. Whiplash Relat. Disord..

[37] Siegmund GP (2011). What occupant kinematics and neuromuscular responses tell us about whiplash injury. Spine.

[38] Staude, G., C. Flachenecker, M. Daumer, and W. Wolf. Onset detection in surface electromyographic signals: a systematic comparison of methods. EURASIP J. Adv. Signal Process., 2001.

[39] Sturzenegger M, Radanov BP, Di Stefano G (1995). The effect of accident mechanisms and initial findings on the long-term course of whiplash injury. J. Neurol..

[40] Stutzig N, Siebert T (2017). Assessment of the h-reflex at two contraction levels before and after fatigue. Scand. J. Med. Sci. Sports.

[41] Stutzig N, Siebert T, Granacher U, Blickhan R (2012). Alteration of synergistic muscle activity following neuromuscular electrical stimulation of one muscle. Brain Behav..

[42] Tomalka, A., C. Rode, J. Schumacher, and T. Siebert. The active force–length relation- ship is invisible during extensive eccentric contractions in skinned skeletal muscle fibres. volume 284, The Royal Society, 2017. 10.1098/rspb.2016.2497 PMC5443931 28469023

[43] Vasavada, A. N., J. R. Brault, and G. P. Siegmund. Musculotendon and fascicle strains in anterior and posterior neck muscles during whiplash injury. Spine 32, 2007. 10.1097/01.brs.0000259058.00460.69 17414909

[44] Weidner, S., A. Tomalka, C. Rode, and T. Siebert. How velocity impacts eccentric force generation of fully activated skinned skeletal muscle fibers in long stretches. J. Appl. Physiol., 2022. 10.1152/japplphysiol.00735.2021 35652830

